# Respiratory Rate Measurement Using Mobile Applications in Healthcare Settings: A Scoping Review

**DOI:** 10.1049/htl2.70035

**Published:** 2026-01-28

**Authors:** Lachlan Sallabank, James Oswald, Sian Willett, James Kelleher, Brian Haskins

**Affiliations:** ^1^ College of Sport, Health and Engineering Victoria University Melbourne Victoria Australia; ^2^ Ambulance Victoria Melbourne Victoria Australia; ^3^ New South Wales Ambulance Sydney NSW Australia

**Keywords:** medical device, patient monitoring, RR, review, vital signs

## Abstract

Respiratory rate (RR) is a strong indicator of clinical trajectory and forms the basis of patient care and assessment. However, clinicians often face barriers to easily obtaining a RR without inefficient methods or costly technology. To remedy this, several phone applications have emerged where clinicians can tap out each breath to calculate a RR. We aimed to map the available evidence for tap‐per‐breath applications used in healthcare settings. We searched for articles using multiple databases, including primary research articles that evaluated tap‐per‐breath apps in healthcare settings. 14 articles were selected for this review, mostly cross‐sectional and hospital based. Most applications reported high usability and efficiency, although results of accuracy were mixed across the included literature. Median‐based apps were more often an accurate measure of RR, however more research is required. Articles were commonly limited in generalisability due to poorly defined reference standards, small sample sizes, or using retrospective video recordings for patient assessment. Studies showed favourable usability and efficiency across the literature, with median‐based apps demonstrating greater consistency and accuracy of RR measurements. Though the scope of this review and limited evidence restrict any far‐reaching clinical implications until further evidence emerges.

## Graphical abstract

Tap‐per‐breath mobile applications estimate RR from breath‐breath intervals on a mobile phone. This scoping review maps 14 primary studies across clinical settings. Usability and efficiency were consistently favourable; accuracy was mixed overall, with median‐based algorithms more often meeting clinically acceptable agreement. Evidence gaps include adult and pre‐hospital use and studies with robust simultaneous reference standards.

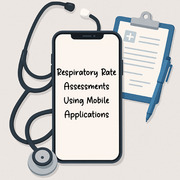

## 1 Introduction

Since the wider adoption of RR in the late 19^th^ century, it has grown to be an influential vital sign with strong evidence for its reliability in predicting clinical deterioration [1]. RR is commonly understood as the number of respiratory (breathing) cycles in a 60 s period. A variety of modalities exist for measuring and estimating RR, ranging from manual counting to advanced automated devices. Manual methods such as 60 s timers or counting beads are easily accessible, although prone to inaccuracy and usability limitations [2]. Automated methods such as capnography or electrocardiogram (ECG) derived RR have gained popularity in recent years, though can be a financial burden for low resource settings. Several researchers have questioned the current methods of manually counting RR [3], with evidence suggesting they are prone to inaccuracy and poor estimation [4, 5]. To address these issues, a more recent method for measuring RR has emerged over the past decade, where clinicians can “tap‐out” each breath on a mobile device, employing an algorithm to calculate a rate per minute. These ‘tap‐per‐breath’ (tap‐bpm) calculators offer a cost‐effective RR alternative, using the time interval between taps to estimate the breaths per minute (bpm). Whilst there is a substantive base of original research evaluating tap‐per‐breath calculators, there remains no comprehensive reviews which map the available literature on this topic. Given the increasing attention from researchers and clinicians, a review is necessary to amalgamate the existing evidence for tap‐per‐breath calculators and direct future research and use. This scoping review aimed to map the available literature on tap‐bpm applications' use and characteristics within healthcare settings.

## 2 Methods

### 2.1 Protocol and Registration

This review's protocol followed the Joanna Briggs Institute (JBI) scoping review methodology [6], and was written in accordance with the PRISMA‐ScR reporting item guidance for scoping reviews [7]. This review's protocol was registered with Open Science Framework (OSF) on the 31^st^ of March 2025 [8]. This research did not directly involve human participants and thus did not require an ethical review.

### 2.2 Eligibility Criteria

This scoping review included studies focused on tap‐per‐breath mobile applications for RR measurement in healthcare settings and primary research articles. We excluded articles not available in English; without full text available; and not meeting the inclusion criteria. This scoping review included all years of publication to ensure relevant papers were not omitted.

### 2.3 Information Sources and Search

The databases and search platforms used for this review included: PubMed, CINAHL, Scopus, and Google Scholar. The most recent search for articles took place on the 19th of March 2025 using the following query strings refined using the Polyglot Search Translator [9]:

*‘(‘respiratory rate’ OR ‘breathing rate’) AND (‘mobile application’ OR ‘tap BPM app’ OR ‘smartphone app’)’*

*‘(‘respiratory rate‘ OR ‘breathing rate’) AND (‘mobile application’ OR ‘smartphone app’ OR ‘digital tool’) AND (‘measurement’ OR ‘monitoring’ OR ‘validation’)’*

### 2.4 Selection of Sources of Evidence

**FIGURE 1:**
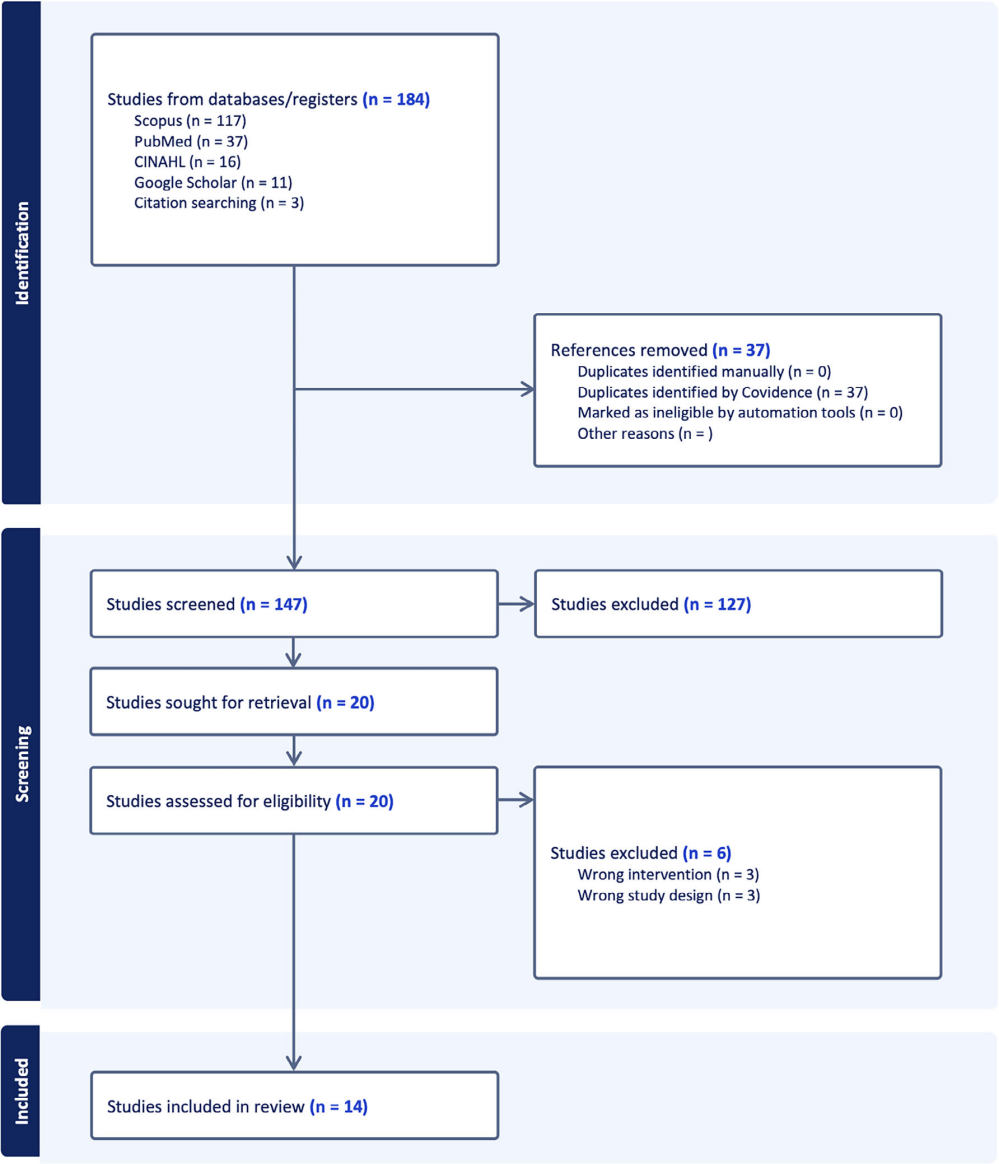
PRISMA flow diagram of the article screening and selection process.

Two independent reviewers (LS, SW) undertook blinded screening of articles using Covidence web app, initially screening titles and abstracts, and proceeding to full text review. Any disagreement with screened articles was resolved by discussion with a third reviewer (JO). A PRISMA 2020 flow diagram was used to track progression through the screening process (Figure 1). Following selection, forward and backward citation tracking was performed to ensure all relevant articles were screened.

### 2.5 Extraction and Synthesis

One author (LS) conducted data extraction of included articles, with extraction checked by the research team for completeness and accuracy. A pre‐determined extraction template was used to collect information including author(s), date of publication, country, conflicts of interest and funding, design, aim, intervention (app) used, RR reference standard, population, outcomes and findings. All extraction took place within the Covidence web application and was later exported to a shared digital spreadsheet. A pilot extraction of five articles was conducted before further collection to ensure functionality and completeness of the extraction template. Given the objectives and scope of this review, no critical appraisal of articles was undertaken by reviewers. Data from included studies were charted and summarised descriptively using a spreadsheet, followed by simple thematic grouping of included studies based on app type, setting, app user, and outcome measures.

Data relating to measures of accuracy, efficiency, and usability were extracted from each study using the authors’ description of the measurement and analytical approaches taken. Accuracy referred to the closeness of tap‐bpm measurement to a pre‐defined reference standard or comparator, usually through Bland‐Altman analysis, LOA, and bias. Efficiency referred to measures of time, or duration of the RR measurement. Usability included any qualitative or quantitative evaluations of user experience in using tap‐bpm apps and devices.

## 3 Results

The search of databases yielded a total of 184 articles, which left 147 articles for screening after removal of duplicates. Title and abstract screening excluded 127 articles based on the inclusion/inclusion criteria, mostly due to incorrect intervention or study design, leaving 20 papers for full text review. Following full‐text screening of 20 papers, 14 were selected for inclusion and review. Of the 147 articles screened for inclusion, three were sourced through backward citation tracking of included articles. The authors used a PRISMA diagram (Figure 1) to track the progression of article selection.

**FIGURE 2:**
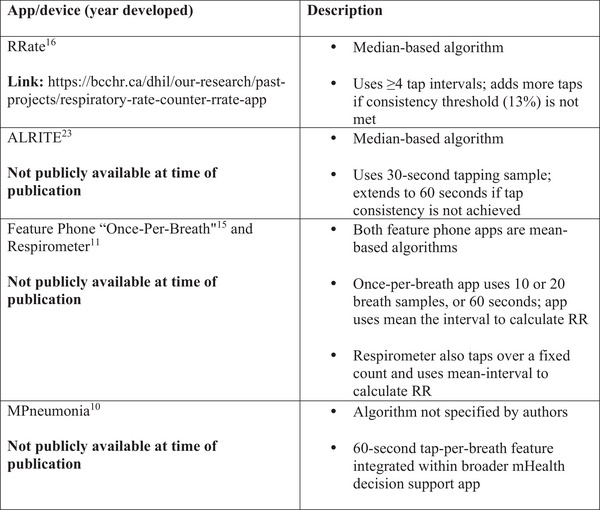
Types of tap‐per‐breath apps available and description of each.

Of the included papers, none were published earlier than 2014, and articles originated from a total of 11 countries. Most of the included studies used observational cross‐sectional study designs, although two secondary data analyses were also included for review. Our review found a total of five tap‐per‐breath applications across four devices, summarised in Figure 2. Table 1 summarises the characteristics of included studies, Table 2 outlines the population characteristics, and Table 3 provides a summary of each article's findings and limitations.

**TABLE 1:** Characteristics of included studies: Study characteristics of articles selected for review.^a^

Author, year, country	Aim	Study design	App/methods used	Reference standard used	Setting
Ginsburg et al., 2015, Ghana [10]	To identify and resolve usability problems of the mPneumonia app within community health clinics in Ghana, and to improve the likelihood of successful integration into practice.	A mixed methods observational design‐stage usability study.	mPneumonia smartphone app, uses 60 s tap‐per‐breath.	Not applicable (study focused on app usability rather than diagnostic accuracy against a gold standard).	Rural health centres in Ghana.
Baker et al., 2019, Cambodia, Ethiopia, South Sudan and Uganda [11]	To evaluate the accuracy of four respiratory rate counting devices used by community health workers detecting pneumonia in children aged 0–59 months in low resource hospital settings.	A Prospective, Multicentre, Hospital‐Based, Single‐Blinded, Comparative Trial.	RRate; Mark Two ARI Timer (MK2 ARI); ARI Timer with counting beads; Respirometer (feature phone app, 10, 20 tap [mean interval], and 60 s count).	Capnography: ISA CO2 nasal capnography (adult size).	Cambodia: Borkeo Hospital Ethiopia: Yrgalem District Hospital South Sudan: Aweil General Hospital Uganda: Mpigi Health Centre IV.
Pöyry et al., 2023, Finland [12]	To evaluate parental ability to assess their child's HR and RR using tap‐per‐breath/tap‐per‐beat mobile applications.	Controlled diagnostic accuracy study.	RRate; ‘BPM tap’ application for HR measurement.	60 s manual count by a healthcare professional for RR.	Pediatric clinic in a secondary care hospital in northern Finland.
Karlen et al., 2015, Switzerland and Canada [13]	To comment on and complement the findings of Black et al. (2015) by comparing the efficiency of various respiratory rate measurement algorithms using a previously published dataset.	Secondary analysis of existing data from a previously published observational study.	Karlen et al.’s algorithm (RRate algorithm) using median of consistent breath intervals (*N* = 4, ThC = 13%); Versus Black et al.’s OPB10Count, OPB20Count, and OPB60Count using mean tap intervals.	Expert consensus X2 (watched videos to reach agreement).^a^	Controlled review of 10 pre‐recorded videos of children breathing (standardised 60s videos).
Asdo et al., 2025, Uganda [14]	To investigate the repeatability of the RRate mobile app.	Secondary analysis of a diagnostic repeatability study within a multisite implementation project.	RRate	No external reference standard; study assessed repeatability by comparing two sequential RRate measurements taken by the same observer under consistent conditions	Outpatient department of Jinja Regional Referral Hospital and Gulu Regional Referral Hospital in Uganda.
Black et al., 2015, Australia [15]	To test whether any of four alternative mobile phone respiratory timer application approaches for feature phones could potentially replace the current standard one‐minute timer approach among professional health workers.	Cross‐sectional diagnostic accuracy and usability study.	Other: once‐per‐breath 10‐breath (OPB10)—mean interval; once‐per‐breath 20‐breath (OPB20)—mean interval; once‐per‐breath 60 s (OPB60)—mean interval; 10‐breath timer (Breath10); Standard 60‐second timer; All apps were Java‐based and tested on Nokia C1‐01 feature phones.	60 s manual breath count.	University‐based study using video recordings in a classroom setting (University of Melbourne, Australia).
Karlen et al., 2014, Canada and Uganda [16]	To investigate if the measurement of breath variability is suitable to assess the recording quality and the confidence in the measured RR.	Observational validation study using both laboratory and field components.	RRate	Lab: Expert consensus X2 (watched videos to reach agreement); Field: No gold standard comparator was used, post‐hoc confidence analysis served as a quality proxy.	Lab: University of British Columbia using standardised videos Field: Level IV rural health centre in Uganda.
Suzuki et al., 2022, Japan [17]	To validate the RRate app for respiratory rate measurement in adults.	Cross sectional study.	RRate	Expert consensus X2: If results did not agree, they watched the video together to reach consensus.	In a teaching hospital, assumed to be in Japan.
Sikakulya et al., 2024, Uganda [18]	To compare respiratory rates measured on admission using a smartphone app (RRate) over seconds versus a piezoelectric device over 15 min, and evaluate their clinical significance and mortality discrimination.	Prospective observational pilot study.	RRate	Comparator: Piezoelectric belt device measuring RR over 15 min; Reference unclear: No formal gold standard, comparison between two methods (no mention of capnography in this study as the standard).	Kitovu Hospital
Nakitende et al., 2020, Uganda [19]	To compare respiratory rate measurements estimated from 15 s of observation with those calculated using the RRate mobile app in acutely ill adult patients.	Prospective observational study	RRate	60 s manual breath count.	Medical ward (46 beds) of St. Joseph's Kitovu Health Care Complex, Masaka, Uganda.
Rimbi et al., 2019, Uganda [20]	To compare respiratory rate measurements over 15 and 30 s with 60 s measurements.	A prospective single centre observational study.	RRate app programmed to estimate RR from both 15 and 30 s periods of 60 sec measurement, not using consistency thresholds, calculated using median tap intervals.	60 s manual breath count	46 bed medical ward of St. Joseph's Kitovu Health Care Complex.
Gan et al., 2015, Canada [21]	To compare accuracy and efficiency of RRate mobile app with the WHO ARI Timer.	A randomised double cross‐over comparative study for diagnostic accuracy and efficiency.	RRate; WHO ARI Timer.	Expert consensus X2 (watched videos to reach agreement).	Conducted in a paediatric ward.
Karlen et al., 2014, Canada [22]	To develop a novel method for measuring RR and to evaluate its efficiency and accuracy.	Prospective observational study using a simulation‐based experimental design	RRate	Expert consensus X2 (watched videos to reach agreement).	British Columbia Children's Hospital, Vancouver, Canada.
Spurr et al., 2022, United States [23]	This study aimed to evaluate the accuracy and usability of the ALRITE RR counter and a commercially available RR counter app, RRate, with a reference standard.	A randomised cross‐sectional observational study.	RRate (parameters used: 12% consistency threshold, 4 tap intervals minimum); ALRITE Note: ALRITE app calculates RR by counting taps for 30s, and continues for 60s if taps are not consistent. Uses median tap interval.	Expert consensus X2 (watched videos to reach agreement).	Videos: Seattle Children's Hospital; Data collection: Unclear where data collection took place, assumed to be also at Seattle Children's Hospital.

Abbreviations: RR = respiratory rate; CHW = community health worker; HCP = healthcare professional.

^a^ “Expert consensus” refers to two or more clinicians independently determining RR from video before reconciling discrepancies.

**TABLE 2:** Population Characteristics: The app users, and population measured for included articles.^a^

Author, year, country	App users^a^	Population measured (e.g. patients)
Ginsburg et al., 2015, Ghana [10]	7 midwives, community health officers, community health nurses, enrolled nurses, field technician; all literate, English‐speaking, mostly female; many first‐time users of touch‐screen devices.	Children presenting to primary health clinics (though focus was on HCP use, not direct patient testing).
Baker et al., 2019, Cambodia, Ethiopia, South Sudan and Uganda [11]	79 community health workers (CHW) trained in pneumonia detection.	454 children aged 0–59 months presenting with cough and/or difficulty breathing.
Pöyry et al., 2023, Finland [12]	203 parents of the paediatric patients.	203 children‐parent pairs aged 0–16 years visiting a paediatric clinic.
Karlen et al., 2015, Switzerland and Canada [13]	22 healthcare‐affiliated researchers or students.	10 children in video recordings breathing at 17–59 breaths/min.
Asdo et al., 2025, Uganda [14]	14 nurses	3679 patients less 5 years of age presenting with acute illness.
Black et al., 2015, Australia [15]	70 student volunteers, mostly nursing students and some physiotherapy.	Five video recordings of children with respiratory illness, with 2 min of running time each.
Karlen et al., 2014, Canada and Uganda [16]	Lab: 22 adult volunteers (presumed healthcare‐related background); Field: Trained healthcare workers using the app as part of routine clinical care.	Lab: Children aged 0–5 years shown in 10 video recordings Field: 322 children aged 0–12 years presenting to a health centre.
Suzuki et al., 2022, Japan [17]	59 nursing students (mean age 20.6 years, 86.4% female).	5 videos of adult patients with visible thoracic respiratory motion (median age 78 years, 20–36 br/min).
Sikakulya et al., 2024, Uganda [18]	Research nurses	403 consenting non‐pregnant acutely ill patients aged 18 year of age or older who were admitted to the hospital's medical ward.
Nakitende et al., 2020, Uganda [19]	2 trained nurses (IN and TN) using Android tablets with RRate; no detailed demographic data provided for observers.	272 acutely ill adult inpatients (mean age 46 years; 52.6% male); 870 recordings collected during hospital admission.
Rimbi et al., 2019, Uganda [20]	2 trained nurses (I.N. and T.N.) performed the measurements using the RRate app.	321 acutely ill adult patients (mean age 49.6 years; 39.3% male; 5.6% in‐hospital mortality); 770 RR recordings.
Gan et al., 2015, Canada [21]	20 adult nursing staff and trainees from BC Children's Hospital.	10 anaesthetised children aged 0–59 months (video), RR range 17–59 br/min.
Karlen et al., 2014, Canada [22]	32 adult users, researchers, med students, engineers, nurses.	10 anaesthetised children aged 0–59 months (video), RR range 17–59 br/min.
Spurr et al., 2022, United States [23]	39 healthcare workers, students and trainees (aged >18, English fluent).	30 children aged 1–24 months admitted with acute lower respiratory infections—videos.

^a^ Several studies included repeated measures per participant; reported sample sizes reflect participants, not the total number of RR recordings.

**TABLE 3:** Results and limitations: The key findings and limitations for the included studies.^a^

Author, year, country	Key findings^a^	Limitations
Ginsburg et al., 2015, Ghana [10]	17 critical and 9 noncritical errors were identified, mostly related to navigation and interface design; All HCPs reported high willingness to use the app with sufficient training (2 days); Preference for mPneumonia over paper‐based protocol was unanimous.	Small sample size (*n* = 7), limiting generalisability; No diagnostic performance evaluation (e.g., sensitivity/specificity); Reliance on self‐reported feedback and short‐term observation; Initial inexperience with touch‐screen technology introduced learning curve.
Baker et al., 2019, Cambodia, Ethiopia, South Sudan and Uganda [11]	Bias vs reference standard was 5.5 bpm for RRate, and 0.5 for Respirometer; Cohen's Kappa for RR classification showed moderate agreement with reference standard at 0.44 for RRate, and 0.41 for Respirometer; Device performance was poorer in infants under 2 months of age; None of the devices demonstrated strong agreement with the reference standard.	Low agreement across all devices, especially limited performance in younger infants; Human counters excluded as reference due to asynchronous timing with devices; Study limited to short‐term hospital settings; real‐world CHW use may differ; Adult nasal capnography used on paediatrics may limit reference reliability; Limited information on whether reference RR was captured simultaneous to new methods, or sequentially, and with what algorithm.
Pöyry et al., 2023, Finland [12]	RR measurement bias for children under 2: 0.8 br/min (SD 9.8; LoA −20 to 19 brpm); RR measurement bias for children 2–16: 0.9 br/min (SD 7.4; LoA −6 to 15 brpm); Sensitivity of subjective parental opinion for recognising tachypnea was 37% (95% CI: 25%–51%); 94.1% of parents were able to attempt at least one RR measurement.	No assessment of how parental education affected accuracy; App versions and technology changed during the study.
Karlen et al., 2015, Switzerland and Canada [13]	Karlen's algorithm achieved 95% of recordings under 19.6s (*N* = 4, ThC = 13%), faster than OPB10Count (35.5 s), OPB20Count, and OPB60Count (60 s); Shorter observation windows can increase efficiency without major loss in accuracy. Using the median instead of the mean helps reduce outlier impact. Manual breath counting is error‐prone and time‐consuming.	Does not compare accuracy of respiratory rate between methods.
Asdo et al., 2025, Uganda [14]	Interrater reliability of 0.95 classified as very high repeatability; 98.9% of measurements completed in <15 s; Strong agreement between measurements with bias of 0.24 breaths per minute; Classification of breathing rate changed between measurements in 12.6% of cases.	Did not evaluate accuracy; Small app user pool; Limited to two institutions; Did not record failed measurements.
Black et al., 2015, Australia [15]	The once‐per‐breath (OPB) 60 s method had the highest reliability, with an ICC of 0.922 and a standard error of measurement (SEM) of 1.10 bpm; The OPB 20‐breath method also performed strongly (ICC = 0.847, SEM = 1.61 bpm), and was noted to be faster for children with elevated respiratory rates; Both OPB methods outperformed the standard 60 s count and significantly outperformed the 10‐breath methods. Limits of agreement (LOA) for OPB methods were narrower (± 10 bpm) compared to other approaches. Participants rated the OPB methods slightly higher in usability, though differences were not statistically significant.	No external gold standard was used; comparisons were made against participants' own 60 s counts; Study used a small number of video clips (*n* = 5) with low variability, may not reflect real clinical variability; Participants had limited time (≈5 min) to familiarise themselves with the apps before testing; Participants were health professional students in a high‐resource setting, not representative of CHWs in LMICs; Use of unfamiliar feature phones (vs smartphones) may have impacted performance.
Karlen et al., 2014, Canada and Uganda [16]	Best performance was seen when measuring three or four breaths with consistency thresholds of 11% and 14%, respectively; Confidence in the results was higher in the lab (around 93%) than in the field (as low as 83%), reflecting more variability in real‐world conditions; The app produced results in 5.8–6.8 s, making it up to six times faster than traditional 60 s counts; Measurement consistency followed a typical statistical pattern but was lower in field use due to clinical challenges.	Only four tap intervals (five taps total) were allowed, limiting opportunities for confident measurements; No direct gold standard (e.g. capnography) was used in field validation; CONF thresholds derived from lab data may be overly conservative for clinical field use.
Suzuki et al., 2022, Japan [17]	Bias (RRate vs reference): +0.40 br/min, LoA −2.86 to +3.67 br/min; RRate mean time to measure: 22.8 s (95% CI: 13.9–36.6), significantly faster than the 60 s method.	No patients with normal RR or bradypnea were involved; Reference videos may not reflect real‐life clinical use; Conducted on nursing students without extensive clinical experience and may not have validity to experienced nurses.
Sikakulya et al., 2024, Uganda [18]	RRate showed a linear increase in mortality with rising RR (unlike pRR which was U‐shaped); Optimal RRate threshold for predicting mortality: 23–26 bpm; Odds ratio for death within 24 h if RRate >26 bpm = 12.22 (95% CI 3.40–44.52); Bland‐Altman bias between NEWS scores = 0.24 points; LOA = –3.03 to 3.51.	Small, single‐centre pilot study; The piezoelectric belt measured over an extended duration, potentially capturing variation not seen in the shorter app‐based assessment; No formal reference standard (e.g., capnography) used for comparison; Convenience sampling may have created a selection bias.
Nakitende et al., 2020, Uganda [19]	Mean RR bias was less in the RRate algorithm (0.11 bpm) than the 15 s method (1.85); RRate correctly assigned NEWS points in 88% of cases vs. 80% for 15 s estimate; RRate required a mean of 6.4 taps (range 5–12) with a mean of 16.2 s (range 4.4–57.6) to compute a reading; Stronger correlation with 60‐second RR for RRate (*r* = 0.91) than for 15‐second observation (*r* = 0.80).	No external reference standard (e.g. capnography) used; Single‐centre study with limited generalisability; Observations over 60 s assumed to be accurate, but are themselves imperfect; Only two participants taking RR measurements.
Rimbi et al., 2019, Uganda [20]	15 s vs 60 s had a bias of 1.22 bpm, LoA = –7.16 to +4.72 bpm; 30s vs 60s had a bias of 0.46 bpm, LoA = –3.89 to +2.97 bpm; 15s method had correlation coefficient of 0.64, whilst 30 s had one of 0.85; Only 25 (51.0%) of NEWS scores were correctly identified by 15s counts; 37 (75.5%) correctly identified by 30 s counts.	Single‐centre observational study, limiting generalisability; Respiratory rates were measured by only two nurses, and inter‐rater reliability was not assessed; No patients had respiratory rates <9 bpm, so NEWS scores for bradypnoea (score of 3) were not evaluated; Correlation was reported, but this does not reflect true agreement between methods; Bland‐Altman analysis was more appropriate; Although the Bland‐Altman method is debated for repeated measures, it is widely used and supported by alternative CI calculations (e.g. Hamilton and Stamey).
Gan et al., 2015, Canada [21]	RRate and ARI Timer both highly correlated with reference (*r* = 0.991 and *r* = 0.982); RRate more accurate with lower percentage error (10.6% vs 14.8%); RRate faster by 52.7 s (95% CI: 50.4–54.9s).	Use of video recordings rather than real patients; Volunteer users may not represent typical clinical usage.
Karlen et al., 2014, Canada [22]	Accuracy of RRate improved with more tap intervals, and exclusion of irregular taps using consistency threshold; Mean time to complete RR estimation: 9.9 s; NRMSE: 5.6%, corresponding to 2.2 breaths/min error at RR = 40 bpm.	Simulated setting only, No live patient measurements; Adult users only: Results may differ with community health workers or less tech‐literate users; Small sample size for Phase II (only 8 nurse participants completed all tasks).
Spurr et al., 2022, United States [23]	ALRITE vs Reference: Spearman's ρ = 0.83 (95% CI: 0.78–0.87), Bias = −2 breaths/min (LoA −16 to +12); RRate vs Reference: Spearman's ρ = 0.62 (95% CI: 0.52–0.70), Bias = −0.4 breaths/min (LoA −24 to +23); ALRITE showed better agreement and usability; 95% of participants rated ALRITE easy to use; Both apps had reduced agreement at higher respiratory rates.	Video measurement may have limited how participants' usually measure RR (tactile or auditory); Low‐moderate generalisability to real world settings; Could not stratify results to participants' clinical experience due to sample size.

^a^ Accuracy, efficiency, and usability are summarised using the authors’ definitions, which varied across studies.

### 3.1 Median Interval Applications

There were two applications found across the literature that calculate the RR based on the median tap interval (measured in seconds), where the tap interval is the time between two taps (breaths). The RRate app developed by Karlen et al. [16]. uses a minimum of four tap intervals to calculate RR and accepts more taps if a consistency threshold of 13% is not met—until four consistent intervals are recorded (using default app settings). The ALRITE app developed by Spurr et al. [23] calculates RR from a 30 s sample of tap intervals, using the median of intervals to estimate the rate per minute. ALRITE also extends the measurement to 60 s if consistent taps are not recorded within the first 30 s.

A total of 12 articles were found to have evaluated RRate and/or ALRITE. Of those which reported accuracy (7 in total), 4 (57%) concluded the app/s had clinically acceptable accuracy; 2 (29%) reported mixed results of accuracy; and 1 (14%) study concluded poor results of accuracy. All six articles which reported efficiency of median‐based apps concluded favourable results with measurement duration being less than the reference or comparator. Similarly, two articles reported usability of median‐based apps, with both concluding that RRate and/or ALRITE had promising usability.

### 3.2 Mean Interval Applications

This review found two tap‐bpm applications across three studies which estimate RR from the mean tap interval [11, 13, 15]. Baker et al. evaluated the respirometer feature phone application which used 10‐tap and 20‐tap option for users. Black et al. also evaluated their own mean‐based feature phone application, similarly using 10 and 20 tap ‘once‐per‐breath’ (OPB) options. Both the Respirometer and the OPB feature phone used the mean tap interval (in seconds) to estimate the breathing rate, rather than the median tap interval.

Two articles assessed the accuracy of mean‐based methods, with mixed results across both. Black et al. found varying results for their OPB app, with the 10‐breath method performing unfavorably compared to a reference standard. However, their 20 and 60‐breath methods both showed high agreement to the reference standard. In contrast, Baker et al. concluded poor accuracy for their tap‐bpm feature phone for RR measurement in children. Black et al. reported good usability of the OPB feature phone application, although it was the only study to report usability for mean‐based apps. Karlen et al. [13] compared the efficiency of the OPB (mean) algorithm to the RRate median‐based algorithm and found that the mean‐based app was less efficient compared to the RRate algorithm and may introduce outlier skewing due to the measure of central tendency.

### 3.3 Other Applications

This review found one tap‐per‐breath application that did not use mean or median‐based algorithms to determine RR (or at least not specified). The mPneumonia app developed by Ginsburg et al. [10] aimed to detect pneumonia in low resource settings, employing a 60 s tap‐per‐breath function to ascertain RRs. Their study primarily focused on usability of the app and found that health workers rated the app more highly than paper‐based protocols. Across all included studies and applications, accuracy results were mostly mixed or favourable, as defined by the authors of included articles in Table 4.

**TABLE 4:** Summary of how accuracy, efficiency, and usability were measured across included studies.

Study author, year and country	Measure of accuracy	Measure of efficiency	Measure of usability
Ginsburg et al., 2015, Ghana [10]	Not reported	Not reported	Usability assessed via task analysis, think‐aloud, and system usability scale.
Baker et al., 2019, Cambodia, Ethiopia, South Sudan and Uganda [11]	Accuracy assessed using mean difference, proportion of readings within ± 2 breaths per minute of the reference, Bland‐Altman analysis, and Cohen's κ, positive percent agreement and negative percent agreement.	Not reported	Not reported
Pöyry et al., 2023, Finland [12]	Accuracy assessed using bias and Bland‐Altman limits of agreement.	Not reported	Usability assessed by percentage of parents able to complete at least one RR measurement.
Karlen et al., 2015, Switzerland and Canada [13]	Not reported	Efficiency explicitly defined as the duration of measurement required to obtain a reliable respiratory rate, comparing RR*ate* to fixed‐breath algorithms.	Not reported
Asdo et al., 2025, Uganda [14]	Not reported	Efficiency defined as time required to obtain a measurement (≤15 s).	Not reported
Black et al., 2015, Australia [15]	Accuracy assessed with Bland‐Altman limits of agreement, intra‐class correlation coefficient (ICC), and standard error of measurement.	Not reported	Usability evaluated through an eight‐item Likert questionnaire on ease of use, learnability, and confidence (1–7 scale), adapted specifically for this study rather than a validated tool.
Karlen et al., 2014, Canada And Uganda [16]	Not reported	Efficiency defined as the time required to obtain a stable reading, optimised through the number of breaths (*N*) and consistency threshold parameters via a cost function balancing error and duration.	Not reported
Suzuki et al., 2022, Japan [17]	Accuracy evaluated using Bland‐Altman bias and limits of agreement, percentage error, and root mean square error (RMSE) less than 3 bpm.	Efficiency was defined as the time required to complete a respiratory rate measurement, comparing the app method to the standard one‐minute visual count.	Not reported
Sikakulya et al., 2024, Uganda [18]	Not reported	Not reported	Not reported
Nakitende et al., 2020, Uganda [19]	Accuracy defined as bias and limits of agreement between RRate and 60 s reference; no explicit threshold or criterion for acceptable bias or agreement specified.	Efficiency defined as the duration required to obtain a valid respiratory rate using the *RRate* algorithm; no threshold or criterion for acceptable measurement time specified.	Not reported
Rimbi et al., 2019, Uganda [20]	Not reported	Not reported	Not reported
Gan et al., 2015, Canada [21]	Accuracy was defined using agreement between RRate and reference values analysed using Pearson correlation, Bland‐Altman bias and limits of agreement, percentage error (PE), and root mean square error (RMSE); a maximum PE of 30% was cited as the recommended threshold for acceptable agreement.	Efficiency was defined as the time required to complete a measurement, reported as median seconds per count and compared statistically between methods	Not reported
Karlen et al., 2014, Canada [22]	Accuracy was defined as the normalized root mean square error (NRMSE) between the *RRate* and a reference standard; Authors used a target of NRMSE ≤ 4 % as the upper limit of acceptable error for optimisation.	Efficiency was defined as the elapsed time (E) from the first tap to reporting a valid respiratory rate, upper acceptable limits for efficiency were set at median (E) ≤ 15 s and 95th‐percentile (Ep95) ≤ 15 s in the cost‐function analysis.	Not reported
Spurr et al., 2022, United States [23]	Accuracy was assessed using Spearman's correlation coefficient, concordance correlation coefficient, and Bland‐Altman bias and limits of agreement. A correlation coefficient greater than 0.7 was a priori defined as indicating high agreement.	Not reported	Usability was defined and measured using the System Usability Scale (SUS), a validated 10‐item questionnaire adapted for mobile health apps.

### 3.4 Population and Settings

The included studies took place in various settings, with most research undertaken within a hospital or healthcare facility (12 studies or 86%), and the remainder within a laboratory or classroom setting (2 studies or 14%). App users also varied across the included articles, with most studies recruiting healthcare professionals (57%), although other research also focused on healthcare students (21%), parents (7%), or a mix of app users (14%). We found that 10 studies (71%) measured respiratory rate on paediatric patients (including both in‐person and video), while 4 studies (29%) involved adult patients as participants. We also found that 6 studies (43%) used video recordings of patients, rather than in‐person RR observations.

### 3.5 Limitations of Included Studies

Our review found recurring limitations across most articles. Many papers raised generalisability as a limitation, frequently due to small sample sizes, video recording use, or single‐centre study designs. The agreement between methods for calculating RR was also raised as a limitation, indicating the challenges for assessing RR accurately across all methods. One possible limitation of mean‐based apps is their susceptibility to outliers, given the mean is sensitive to skew from outliers. Some authors also raised the lack of a robust reference standard for RR measurement, with many studies also highlighting this as a key limitation. A limitation identified in this review related to how accuracy of tap‐bpm apps was reported in the literature. Many authors chose to use bias and LOA as measures of accuracy; however, bias reflects only the average directional error and can obscure results when positive and negative discrepancies cancel each other out. Our review also revealed that many studies did not assess user training and competence in using tap‐bpm apps, which may have limited findings in some cases.

### 3.6 Measures of Accuracy, Efficiency, and Usability

Pictured above, Table 4 outlines the various methods used across studies to determine accuracy, efficiency, and usability.

## 4 Discussion

This review is the first known to systematically map the evidence surrounding tap‐per‐breath RR applications. Three key findings emerged. First, tap‐per‐breath applications have shown promising usability and efficiency across a range of users and clinical contexts. Second, whilst the accuracy varied between studies, median‐based apps generally performed consistently in measuring RR. Third, the majority of existing research focuses on paediatric patients, in‐hospital settings, and through retrospective video analysis, highlighting the need for more robust and real‐world research.

Several limitations of this review were apparent and worthy of discussion. We excluded non‐primary research in this review to maintain a focus on empirical evidence, although this limited any broader contextual insights. Given the scope of our review, no critical appraisal of articles was undertaken, and thus limited definitive clinical implications. Further to this, this review did not employ a meta‐analytic approach and therefore could not quantify effect sizes or demonstrate statistical significance for the included literature. Notwithstanding these limitations, this first review of tap‐bpm apps revealed an emerging area of clinical interest with a strong potential for further development.

Whilst median‐based apps such as RRate showed mixed accuracy, we hypothesise that substantial heterogeneity may account for these differences. As contended by Ansermino et al. [24] in their response to a study from Spurr et al. [23], it is possible that using breath counting as a reference standard is inadequate given the variance of breath‐to‐breath intervals across an entire minute. It was apparent from this review that those median‐based studies that reported mixed or poor accuracy all used real‐time 60 s counts as a reference standard—and it was often unclear if measurements were simultaneous or sequential. We would like to highlight this as a significant confounder to how tap‐bpm apps are evaluated, and it is likely that accuracy results would improve if more precise reference standards were used.

Despite the increasing relevance of new RR devices, much debate exists over what an appropriate reference standard could be for such studies. Much of the recent research has elected to use video recordings and expert panel review to determine the RR reference standard [25]. However still, video recordings fail to emulate the real‐world clinical environment. The authors of this review echo the sentiment of Ansermino et al. [24, 26] in that reference standards must be clearly defined and methodologically sound—using techniques that allow precise detection of each breath. This may include nasal capnography with timestamps for each breath, expert panel review of video, or even healthy participants breathing to a metronome rate. The latter is a method not yet used in assessing tap‐bpm devices—however, enables a much more realistic environment, which potentially reliable reference standardisation. As for the viability to test these devices in real clinical settings—it is unlikely to yield reliable results of accuracy, but rather worthy of examining other real‐world outcomes for the use of these apps. Such research could evaluate the large scale impact of introducing tap‐bpm technology, potentially analysing whether frequency and distribution of RR values differs over time.

There is an abundant need for future research into tap‐per‐breath applications to better understand their efficacy in clinical settings. Further research should include head‐to‐head validation studies that emulate real‐world practice, in a variety of settings. Given the gap in existing literature, this could include prehospital and austere settings. Consideration should be given to other uses of tap‐bpm apps, particularly in mass casualty triage or newborn heart rate auscultation. As discussed in the limitations of included studies, it is imperative that a robust reference standard for RR is established, which reliably captures the inter‐breath intervals simultaneously to app usage. Moreover, many included studies used bias as a measure of accuracy; however, bias only captures the average directional error between the app and reference standard. This can obscure substantial discrepancies when positive and negative errors cancel each other out. To properly assess the accuracy of tap‐bpm apps, researchers should utilise statistical tests that capture the average error of measurement, regardless of being positive or negative.

Whilst limited, this scoping review has some important implications for clinical practice. The current evidence suggests that median‐based tap‐per‐breath apps are worthy of further development, exploration, and research. Implementation of tap‐bpm apps may be considered for resource limited settings such as remote or prehospital environments, where capnography or other robust methods of RR measurement are not readily available. Given that some clinicians may prefer avoiding mobile phone contact during clinical care, it is worth considering smartwatch implementation, or keychain‐tap devices that could be more usable. However, the scope of this review, and heterogeneity of included studies limit the widespread endorsement of tap‐bpm apps until more robust evidence emerges.

To conclude, whilst the existing evidence for tap‐per‐breath RR apps is limited, this first‐time review revealed promising results for its development and use. To alleviate the challenges of measuring RR in healthcare settings, tap‐per‐breath apps offer a low‐cost alternative with strong results of efficiency, usability, and in some cases, accuracy. Median‐based tap‐per‐breath apps out‐performed mean‐derived apps across the majority of included articles. Further validation is needed before widespread implementation — however, tap‐bpm apps may offer a viable alternative where advanced measurement technology is unavailable. Future studies should focus on real‐world use in prehospital and adult settings, with a focus on providing a reliable reference standard for RR comparison. Healthcare providers should consider tap‐bpm apps for development within existing frameworks and encourage future research to more frequently and accurately measure RR.

## Author Contributions

**Lachlan Sallabank**: conceptualization, data curation, formal analysis, investigation, methodology, project administration, writing – original draft, writing – review and editing. **James Oswald**: conceptualization, investigation, methodology, writing – review and editing. **Sian Willett**: investigation, methodology, writing – review and editing. **James Kelleher**: methodology, writing—review and editing. **Brian Haskins**: conceptualization, investigation, methodology, supervision, writing—review and editing. All authors meet ICMJE authorship criteria, approved the final manuscript, and agree to be accountable for all aspects of the work.

## Funding

The authors have nothing to report.

## Ethics Statement

The authors have nothing to report.

## Conflicts of Interest

The authors declare no conflicts of interest.

## Data Availability

All data is available in either the published articles included in the review, or tables attached to this review.
